# Dexamethasone-boosted mesenchymal stem cell secretome: insight into hepatic protection

**DOI:** 10.1186/s12896-025-00980-8

**Published:** 2025-06-04

**Authors:** Eiman M. Adly, Thoria Diab, Mohamed Hessien

**Affiliations:** Biochemistry and Molecular Cell Biology Unit, Division of Biochemistry, Faculty of Science, Tanta University, Tanta, 31527 Egypt

**Keywords:** MSC-conditioned media, Osteogenesis, Dexamethasone, Acute liver failure, Hepatocellular carcinoma

## Abstract

As the independent anti-inflammatory effects of mesenchymal stem cells (MSCs) and glucocorticoids are well documented, it is hypothesized that the conditioned media derived from dexamethasone (DEXA)-treated MSCs may exhibit a potent therapeutic effect. To explore this, bone marrow-derived MSCs were transiently maintained in DEXA-containing media, where cell viability, phenotype, and osteogenic differentiation were assessed. Furthermore, the MSC-conditioned media (MSC-CN) was utilized to inhibit the proliferation of hepatoma cells and treat drug-induced acute liver failure (ALF) in mice. We found that low doses of DEXA (≤100 nM) maintained MSC viability and their typical mesenchymal phenotype. Conversely, 1000 nM decreased the expression of the mesenchymal markers (CD105 and CD90), triggered osteogenic differentiation as evidenced by the modulation of osteogenesis-related genes (alkaline phosphatase, osteopontin, and Runt-related transcription factor 2), and increased the intracellular calcium, assessed by Alizarin Red S staining. Moreover, MSC-DEXA-S restricted colony formation, cell migration, and glucose consumption in hepatoma cells. In parallel, MSC-DEXA-S protected mice against acetaminophen-induced ALF, where both liver functions, oxidative stress (Nrf-2, SOD1, GSH, and MDA), angiogenic (VEGF), and inflammatory (TNF-α) markers were improved. Also, MSC-DEXA-S resolved liver necrosis one week after transfusion. These data suggest that pretreatment of MSCs with low doses of dexamethasone maintains their stemness and enhances their paracrine therapeutic effect against hepatic diseases.

**Graphical Abstract:**

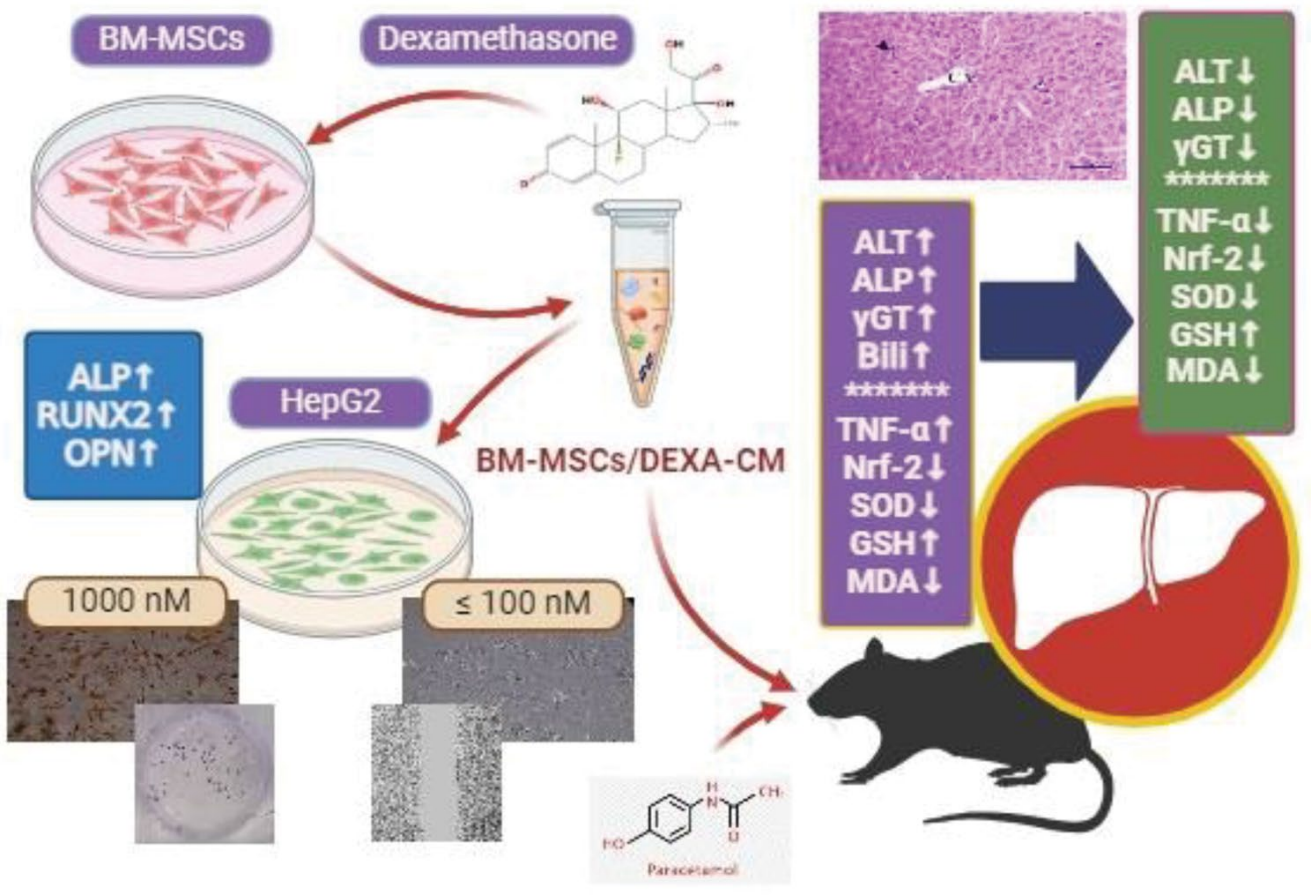

## Introduction

Liver diseases are a major global burden, where they account for two million deaths per year, representing about 4% of the total disease-related mortality [1]. Also, they demonstrate a wide range of pathogenesis, starting from acute liver failure (ALF, also called fulminant hepatitis) to hepatocellular carcinoma (HCC). Liver-related mortality is attributed to rapid and acute hepatitis (ALF) or the slow-growing complications associated with liver cirrhosis and HCC. Commonly, these pathological conditions have a wide range of etiologies including drug-overdosing, xenobiotic, hepatotropic viruses, alcohol, autoimmune disorders [2], and lipid-related metabolic dysregulation [3]. Acute liver failure, for example, is a potentially fatal condition characterized by massive inflammation, rapid injury of liver parenchymal cells, deterioration of hepatic functions, and hepatocellular necrosis [4]. Although the management of liver diseases differs depending on the disease pattern, severity, etiology, and patient-related factors, it starts with removing the causes, such as alcohol withdrawal, hepatitis antiviral drugs, and termination of hepatotoxic medications [5]. Furthermore, advanced cases, particularly cirrhosis and HCC, involve a multidisciplinary approach and collaboration among surgery, oncology, radiology, and pathology to provide optimal outcomes [6, 7]. As the cost-effective treatment strategies for liver diseases are limited, regenerative therapy was introduced, where many clinical trials showed that stem cells might be a promising therapeutic approach[8]. Recently, growing numbers of studies have used various types of stem cells as a valuable tool to cure ALF, cirrhosis, and HCC. The basic concept of stem cell therapy relies on its stemness-related characteristics, differentiation potentials, and paracrine effect. Mesenchymal stem cells (MSCs), in particular, are the most commonly utilized, due to their relative availability, less ethical restrictions, low immunogenicity [9], and anti-inflammatory role [10]. According to previous studies, MSCs may cure liver disorders via their differentiation into hepatocyte-like cells (HLCs), modulating immune cell activity, and/or paracrine healing effects via the biologically active molecules they secrete [11, 12]. However, MSC transplantation has not always been successful as the transplanted cells are challenged by *in vivo* adverse conditions. Additionally, the literature has accumulated many side effects associated with MSC transplantation [13]. Accordingly, many preclinical trials aimed to improve MSC therapeutic potency via preconditioning with physical or pharmacological modulators [14]. In this regard, some authors have combined MSCs with glucocorticoids (GCs), like dexamethasone (DEXA), to avoid transplantation-associated inflammation or enhance MSC differentiation [15, 16]. Although the anti-inflammatory role of DEXA is well reported, many investigations revealed its concentration-dependent pleiotropic effects on MSC fate. Incubation of MSCs with high doses of DEXA, for example, inhibited their growth, meanwhile, lower doses and short-term exposure enhanced their proliferation [17, 18]. These contradictions are attributed to the role of DEXA in regulating genes involved in cell proliferation [19] or mitogenic signaling pathways [20] Although the independent treatment of some liver diseases with DEXA or MSCs was previously investigated [21], the boundaries of DEXA-MSCs co-treatment are not yet resolved. Also, the direct impact of DEXA on the MSC paracrine effect was inadequately addressed. Therefore, this work adopted two approaches. The first was to explore how far exposure of MSCs to different concentrations of DEXA can affect their stemness markers, viability, and osteogenic differentiation. Second, investigate the efficacy of the conditioned media of DEXA-pretreated MSCs against the proliferation and migration of hepatoma cells in culture conditions and treatment of acetaminophen (APAP)-induced ALF in a mouse model.

## Materials and Methods

### Key reagents

Dexamethasone (4 mg/ml) was obtained from Amriya for Pharmaceutical Industries, Alexandria, Egypt. Acetaminophen, 4′-Hydroxyacetanilide (APAP) (CAS No.103–90-2) was from Sigma (St. Louis, USA). Cell culture reagents DMEM, MEM-α with nucleosides, fetal bovine serum (FBS), and penicillin/streptomycin were from Lonza, Switzerland. Phycoerythrin (PE)-conjugated monoclonal antibodies against normal pattern mesenchymal markers (CD105, CD90, CD73, CD45, and CD34) were from Becton Dickinson (San Diego, CA, USA). Annexin-V kit for apoptosis was from Beckman Coulter. Alkaline phosphatase (ALP, E.C.3.1.3.1, Cat No. 215 001), Alanine aminotransferase (ALT, E.C.2.6.1.2., Cat No. 265001), γ-Glutamyltransferase (γGT, E.C.2.3.2.2, Cat No. 247 001), total bilirubin (Cat No. 225 001), and glucose (Cat No. 250 001) kits were from Spectrum Diagnostics, Egypt. Alizarin Red R was purchased from Oxford Fine Lab Chemicals, India.

### Cell line, BM-MSC isolation, and treatments

Hepatoma cell line (HepG2) was obtained from Vascira, Cairo, Egypt. BM-MSCs were isolated from a 12-week-old (250 g) adult male Sprague-Dawley rat, following the ethical regulations of the Faculty of Science, Tanta University Animal Care and Use Committee (IACUC-SCI-TU-0184). Isolation of BM-MSCs was performed as described previously [22]. Briefly, bone marrow, from the tibia and femur bones, was flushed with 10 ml complete media and seeded in MEM-$\alpha$ medium, containing heat-inactivated FBS (10%), and penicillin/streptomycin (1%). Cells were incubated at 37 °C, 5% CO_2_, and saturated humidity, grown to subconfluency (~90%), collected with 0.25% trypsin/EDTA, passaged to the fourth passage, and treated with 10, 100, or 1000 nM DEXA for 24 h.

### Phenotypical characterization of BM-MSCs

DEXA-treated MSCs were investigated for their mesenchymal characteristics. To establish this, 10^6^ cells/ml were incubated for 30 minutes with monoclonal antibodies of different CD markers. The fluorescently labeled cells were analyzed by FACSscan flow cytometer (Becton-Dickinson, Franklin Lakes, NJ, USA), and data were retrieved using CELLQuest Pro (Becton-Dickinson).

### Annexin V-FITC apoptosis assay

The effect of DEXA treatment on BM-MSC viability and possible apoptosis-mediated cell death was determined, as previously described [23], using Annexin V-FITC and propidium iodide (PI) dual staining. The percentages of viable, dead, and apoptotic cells were determined. Briefly, cells were collected, washed with PBS, and suspended in the binding buffer (500 μl). Next, cells were incubated for 15 min at 4 °C with 5 μl Annexin V-FITC and then incubated for an additional 5 min with 10 μl PI. Both green and red fluorescence of Annexin-V, and PI, respectively, were measured by a flow cytometer. Viable, apoptotic, or necrotic cell populations were determined as the percentage of Annexin-V^+^/PI^−^, Annexin-V^+^/PI^+^, or Annexin-V^−^/PI^+^ cells, respectively.

### Assessment of osteogenic differentiation

Alizarin Red-S calcium-specific staining was employed to assess the accumulation of cellular calcium in DEXA-treated BM-MSCs. Cells were cultured at low density in a 6-well plate and incubated overnight at 5% CO_2_. Next, cells were left untreated or treated with 10, 100, or 1000 nM DEXA for 24 h. After discarding the old media, 1 ml of 15% formaldehyde was added per well for 10 min. After fixation, cells were washed with PBS, 1 ml Alizarin Red S (2%, pH 4.1–4.3) was added for 15 min, the dye was removed, and cells were washed with water before imaging under a phase contrast microscope.

### Alkaline phosphatase and glucose uptake measurements

The enzymatic activity of ALP was estimated in lysed MSCs, using *p*-nitrophenyl phosphate as substrate [24]. Briefly, adherent cells were washed with Dulbecco’s PBS, collected by scraping, lysed by sonication, and centrifuged. The ALP activity was determined in the supernatant following the manufacturer’s guidelines. Also, glucose consumption was determined in the culture media of HepG2 cells grown in phenol red-free media using the glucose oxidase (GOD) method, following the manufacturer’s instructions.

### Preparation of DEXA-treated MSC conditioned media

BM-MSC conditioned media (BM-MSC-CM) was prepared by incubating DEXA-treated cells in FBS-free media. After 24 h, media were collected and utilized to investigate the BM-MSC paracrine effect against hepatoma cells or the treatment of ALF mouse model.

### In vitro cell migration assay

Investigating the effect of DEXA-MSC-S on cancer cell migration was performed by Wound healing assay. Initially, cancer cells were cultured in a 12-well plate and left to subconfluency. After a scratch was made in the cell monolayer, cells were washed well with PBS, and incubated in DEXA-treated-MSC-S. The cell-free area was measured after 48 h, using Image J, and compared with the zero-time area.

### Clonogenic assay

To study the effect of DEXA-treated MSC-S on the proliferation of HepG2 cells, a clonogenic assay was performed in a 6-well plate as previously described [25]. Cancer cells were cultured at a low density (500 cells/well) and incubated for 24 h, after which media were replaced with a conditioned media derived from DEXA-treated BM-MSCs. Cells were incubated for 8–10 days, fixed with ethanol (70%), and stained with crystal violet (0.2% dissolved in PBS). Next, blue colonies were counted using Image J.

### Detection of cellular ROS by DCFH-DA and fluorescent microscopy

To investigate the oxidative stress status via the overproduction of cellular ROS, cancer cells (5×10^5^ cells/well) were incubated with DEXA-MSC-S for 48 h. After washing with DMEM, cells were stained with 10 µM (in 500 µl) 2′,7′-Dichlorofluorescein diacetate (DCFH-DA) per well and the plate was incubated in a CO_2_ incubator for 30 min after which the DCFH-DA was decanted and cells were washed twice with DMEM, and once with PBS. Next, 500 µl of PBS was added per well and the cells were examined under an inverted fluorescence microscope (Zoe, BioRAD).

### Gene expression analysis

After cell treatment, total RNA was isolated by RNA-extraction kit (GeneDireX, Inc.). RNA integrity was verified by formaldehyde gel electrophoresis and measuring the OD260/OD280 nm ratio. For the first strand cDNA synthesis, 500 μg RNA was incorporated in RT reaction using 2.5 U reverse transcriptase, 10 U RNase inhibitor, 1 mM dNTP mixture, 1.25 pmol Oligo-dT primer, 5 mM MgCl_2_, and 1 × RT buffer mixed in 20 µl reaction volume. Strips were incubated at 42 °C for 30 min, at 95 °C for 5 min, and at 5 °C for 5 min. qRT-PCR was performed to assess the expression of BM-MSC osteogenic-related genes including Alkaline phosphatase (ALP), Runt-related transcription factor 2 (Runx2), and osteopontin (OPN), using the previously designed primers [26] (Table 1). In the amplification reaction, 25 μg of cDNA was incorporated as a template in a reaction mix containing SYBR-green master mix and 0.2 μM of gene-specific primer pairs. Thermal cycling reactions were performed in QuantStodio 5 (ThermoFisher). The thermocycler program was one cycle at 95 °C for 10 min followed by 40 cycles at 95 °C for 15 s, at 55 °C for 15 s, and at 72 °C for 30 s. GAPDH was included as an internal control. Reactions were performed in triplicate, and the results were analyzed using the previously described [27].

**Table 1:** Sequence of primers used in the expression analysis of osteogenesis-related genes and GAPDH

Genes	Primer sequence
GAPDH	For 5’- AAGGTCATCCCAGAGCTGAA-3’
	Rev 5’- AGGAGACAACCTGGTCCTCA-3’
RUNX2	For 5’- CCGCACGACAACCGCACCAT-3’
	Rev 5’- CGCTCCGGCCCACAAATCTC-3’
ALP	For 5’- AACCCAGACACAAGCATTCC-3’
	Rev 5’- GCCTTTGAGGTTTTTGGTCA-3’
OPN	For 5’-GACGATGATGATGACGATGG-3’
	Rev 5’- CCTCAGTCCATAAGCCAAGC-3’

*For: Forward, Rev: Reverse*

### Induction of acute liver failure and treatment with DEXA-modified MSC-S

**Fig. 1:**
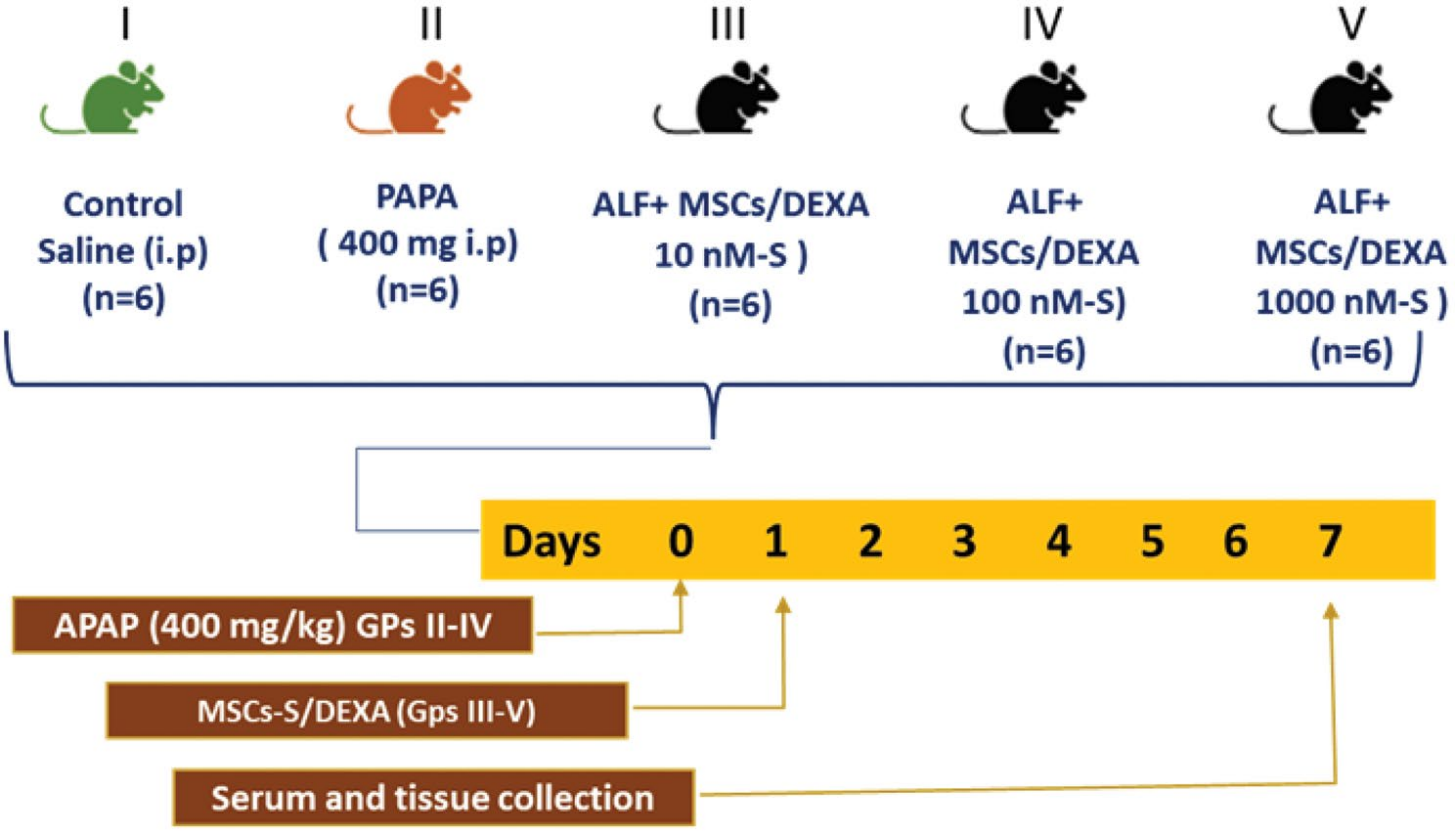
Experimental design, animal grouping, and treatments

Male mice (C57BL/6, 25–31 g body weight) were acclimatized for one week before the induction, had access to food and water, and were housed at 25 ± 1 °C, and a normal light/dark cycle. Previous studies indicated that females may exhibit greater vulnerability to drug toxicity and demonstrate more adverse drug reactions compared to males, due to variations in pharmacokinetics, pharmacodynamics, and hormonal influences. This explains the limitation of using male mice. All mice received care in compliance with the guidelines for the care and use of laboratory animals. Also, the working protocol was approved by the Ethical Committee of the Faculty of Science, Tanta University (IACUC-SCI-TU-0184). Thirty male mice were randomly assigned to five groups, six mice each (Fig. 1). Group I mice received saline, as a control group. Meanwhile, mice in groups II through V received a single intraperitoneal (i.p.) dose (400 mg/kg body weight) of APAP. Group II mice were sacrificed 24 h after APAP injection, meanwhile, groups III, IV, and V were infused with 200 µl of prefiltered MSC-S derived from BM-MSCs pretreated with (10 nM) 10^-8^ M, (100 nM) 10^-7^ M or 10^-6^ M (100 nM) DEXA, respectively via tail vein. Animals were sacrificed one week after infusion, and both blood and liver tissues were recovered for further analysis.

### Serum and hepatic markers

The activity of ALT, ALP, GGT, and the level of bilirubin were assessed in serum samples with marker-specific kits, following the manufacturer’s instructions. Also, liver tissues were homogenized in ice-cold 150 mM phosphate buffer, pH 7.2, containing 1 mM EDTANa2. The homogenate was centrifuged at 15,000 g at 4 °C for 15 min to obtain supernatant. Protein concentrations were estimated by Bradford assay. TNF-α and Nrf2 were determined by mouse TNF-α and Nrf2 ELISA kits (CUSABIO Cat. No. CSB-E04741m, and CSB-E16188m, respectively). VEGF and GSH were determined by marker-specific Elabscience kits (Cat. No. E–EL-M1292 and E–BC-K030-M, respectively). SOD was estimated by RayBiotech SOD kit, following the manufacturer’s guidelines.

### Histopathological and immunohistochemistry analysis

Liver tissue samples were harvested and fixed in buffered formalin (10%). After dehydration and clearance, tissues were embedded in paraffin, sectioned in 5 µm, and stained with hematoxylin-eosin (H&E), following the standard staining protocol. Slides were examined under a light microscope to determine the degree of fibrosis development or regression. Sections were graded from 0 to 4 according to modified Suzuki score as follow: score 0 = no lesion, score 1 = minimal lesion involving less than 25% of liver tissue, score 2 = mild lesion involving 26%– 50% of liver tissue, score 3 refers to moderate lesion involving 51% - 75% of liver tissue and score 4 = severe lesions involving more than 76% of liver tissue (Sabry et al., 2019).

Also, immunohistochemistry was conducted to assess p53 and Bcl2 expressions, where tissue sections (3–5 μm) were deparaffinized, rehydrated, and subjected to antigen retrieval using citrate buffer (pH 6.0). Endogenous peroxidase was blocked with H2O2 (3%). Sections were incubated with P53- or BCL2-specific primary antibodies (Dako North America Inc., Carpinteria, CA, USA) at 4 °C overnight or for 1 h at room temperature, respectively. After washing, rabbit anti-mouse HRP for P53 or biotinylated goat anti-polyvalent with streptavidin-peroxidase for Bcl2 were applied, followed by DAB for colour development. Slides were counterstained with Mayer’s hematoxylin, dehydrated, cleared in xylene, and mounted.

### Statistical analysis and software

Statistical analysis was performed using SPSS software (version 26.0, Chicago, IL, USA). Parametric data are expressed as mean ± standard deviation (SD), where P < 0.05 was considered significant. Data was bar graphed by Microsoft Excel. Image J (http://WWW.Fiji.com) was used to estimate the red stain in the Alizarin Red S osteogenic detection, area measurement in the wound healing assay, and colony count in the clonogenic assay. The target prediction sites of TargetNet (http://targetnet.scbdd.com/) and the Similarity Ensemble Approach (SEA) (http://sea.bkslab.org) were used to predict cellular targets of DEXA and its hydroxylated metabolite.

## Results

### Isolation, characterization, and treatment of BM-MSCs

**Fig. 2:**
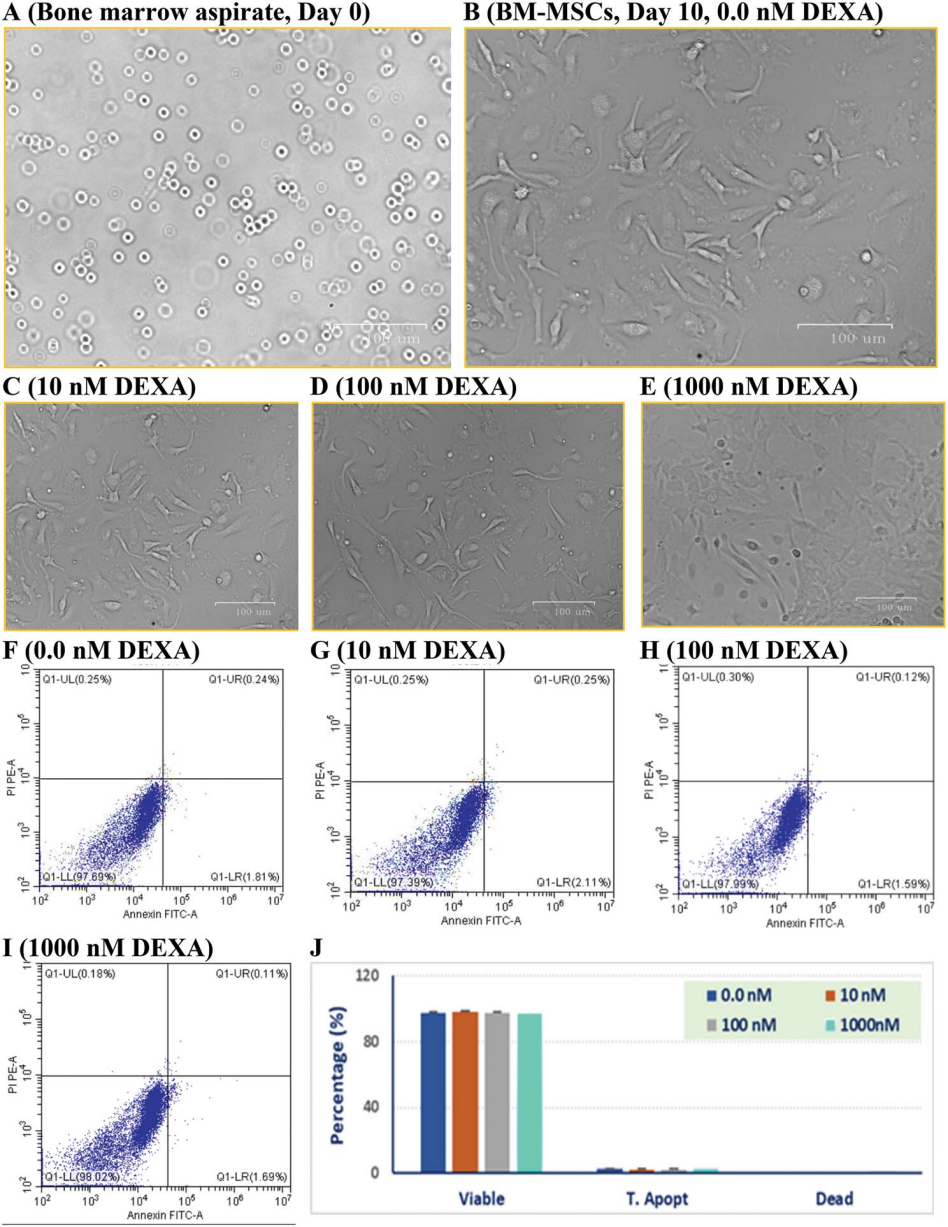
Cellular morphology and viability of BM-MSCs after being treated with dexamethasone. Phase contrast micrographs showing cells aspirated from the tibia and femur of a rat (**A**), cells propagated to the 4^th^ passage (**B**), and cells treated with 10 nM, 100 nM, or 1000 nM DEXA for 24 hours. Cells appeared adherent with a spindle fibroblast-like shape without signs of apoptosis. Cells treated with DEXA maintained their viability. Scatter bolts represent DMSO-treated cells (**F**), cells treated with 10 nM (**G**), 100 nM (**H**) or 1000 nM (**I**) DEXA. Viable, early apoptotic, late apoptotic, and necrotic cells are represented in the lower left, lower right, upper right, and upper left quadrants, respectively. The bar graph (**J**) represents the average percentages of viable, apoptotic, and dead cells, where the drug did not induce significant changes in cell viability compared to untreated cells

**Fig. 3:**
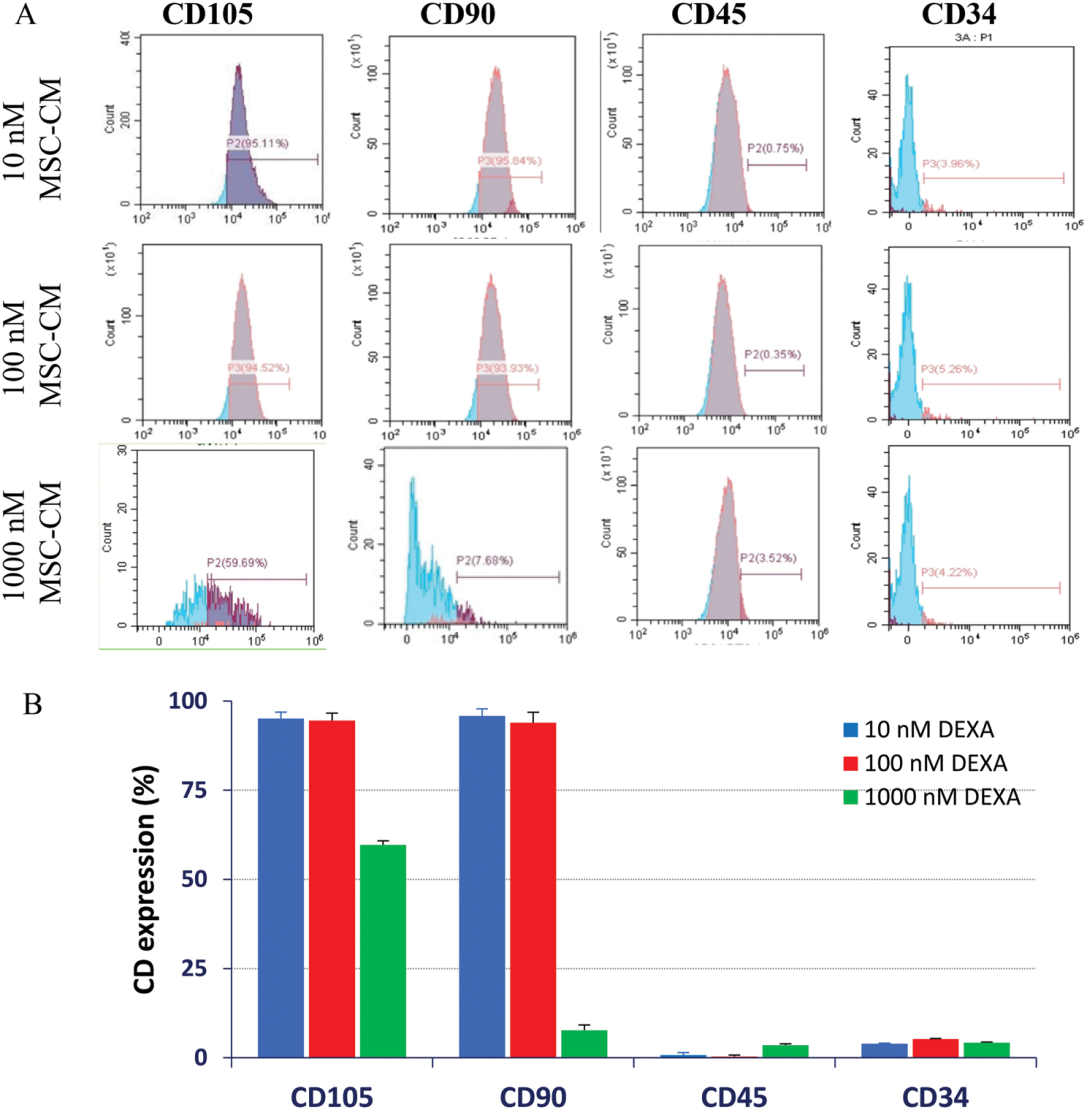
Effect of DEXA on the expression of mesenchymal-specific surface markers. Treatment of MSCs with 10 or 100 nM DEXA did not affect their regular phenotypical characteristics. A significant decrease in the expression of CD105 and CD90 was seen in cells treated with 1000 nM DEXA

Bone marrow was aspirated from the rat’s bone (Fig. 2A), where culture BM-MSCs exhibited a typical spindle and myofibroblast-like shape (Fig. 2B). Exposure of the 4^th^ passage cells to 10, 100, or 1000 nM DEXA for 24 h did not affect cell morphology under a phase-contrast microscope (Fig. 2C-2E). Also, DEXA treatments (with 10, 100, or 1000 nM) did not induce significant apoptotic or necrotic changes, as the percentages of viable cells were 97.39%, 97.99%, and 98.02%, respectively, compared to 97.69% in DMSO-treated control cells (Fig. 2 F-2I, respectively). Additionally, DEXA-treated cells were stained and analyzed for the mesenchymal (CD105, CD90) and hematopoietic (CD45, CD34) surface markers. Cells treated with 10 or 100 nM DEXA were highly expressing CD105 and CD90, (>90%), but minimally (<10%) expressing the hematopoietic markers (CD34 and CD45), evidencing their pure mesenchymal phenotype. However, higher concentration (1000 nM DEXA) caused a significant (P < 0.001) decrease in expression of CD105 (59.69%) and CD90 (7.68%) (Fig. 3A and 3B).

### Assessment of the osteogenic differentiation

**Fig. 4:**
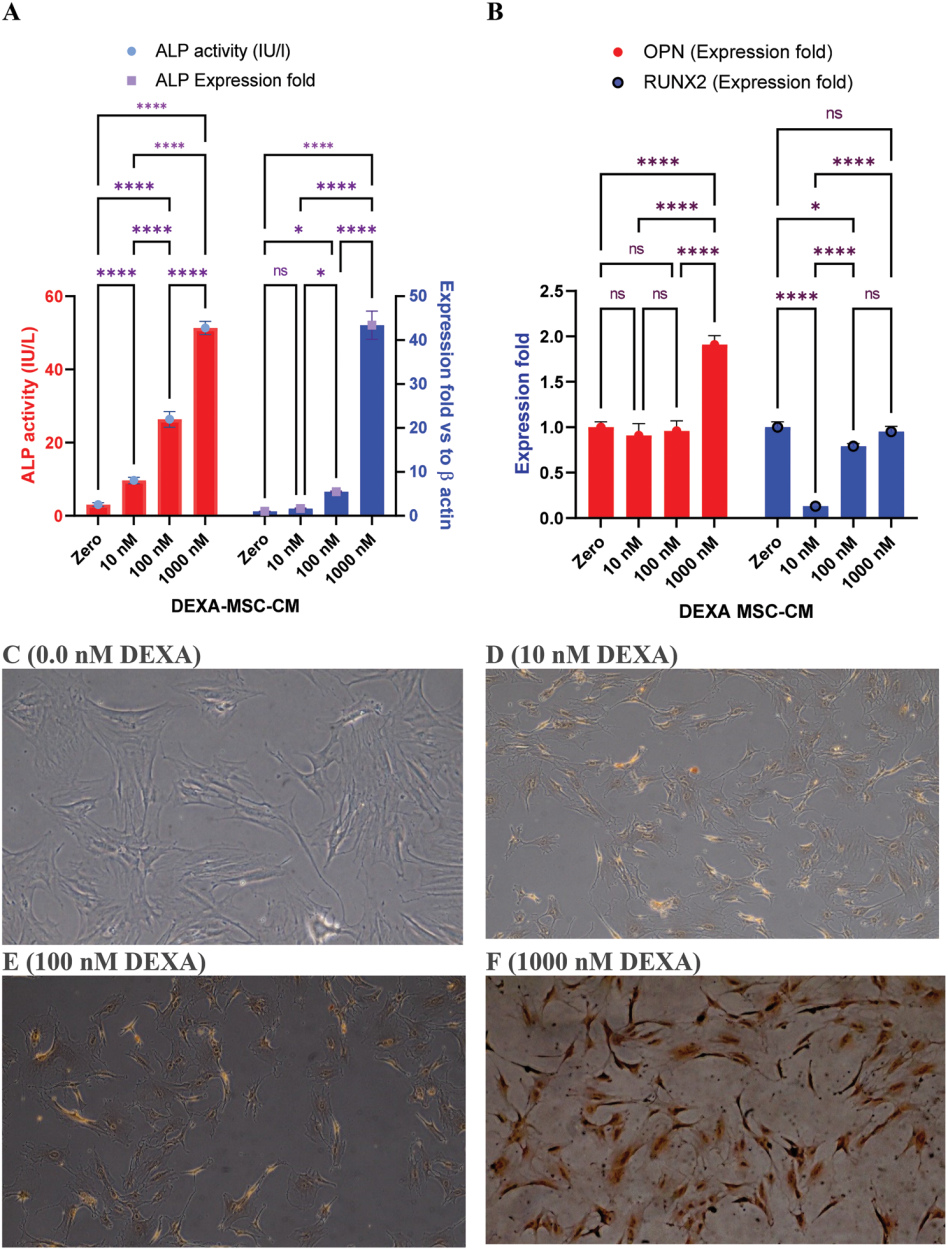
Assessment of DEXA-mediated osteogenic differentiation of BM-MSCs. Effect of DEXA on the activity of ALP, the expression of ALP (B), osteopontin (OPN) (C), and RUNX2 (D) assessed by qRT-PCT. “E” through “H” represents Alizarin S Red staining to assess intracellular calcium deposition after cells were treated with 10 nM (F), 100 nM (G), or 1000 nM (H). Higher doses (1000 nM) induced significant calcium deposition indicating mesenchymal-osteogenic transition. (Magnification 40x). Bars represent means (±SD). (_*_): p < 0.05, and (_***_) p < 0.001 compared to the untreated cells

**Fig. 5:**
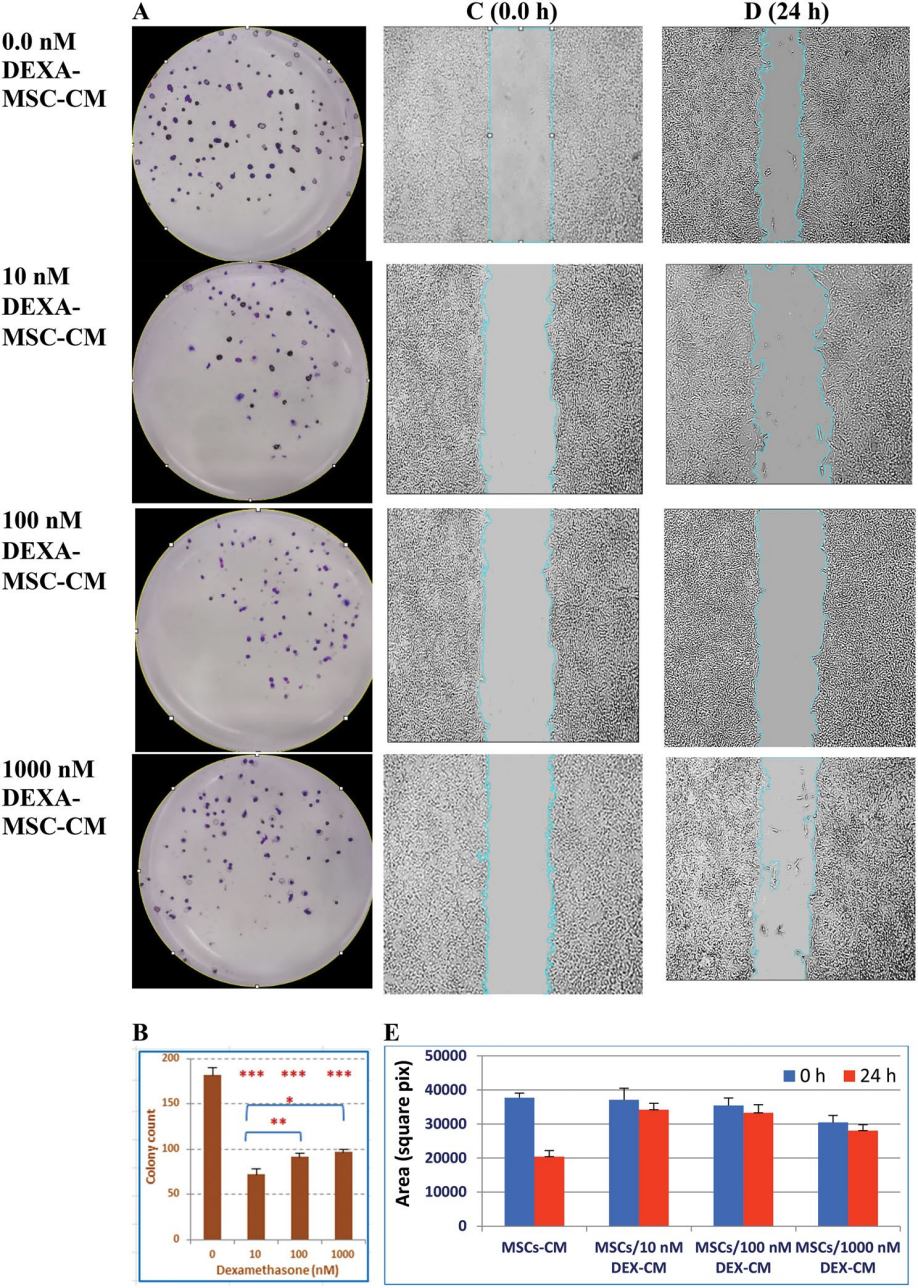
Clonogenic and *in vitro* cell migration assays of hepatoma cells treated with MSC/DEXA-S. HepG2 cells were cultured for 10 days in conditioned media derived from untreated MSCs or cells treated with 10, 100, or 1000 nM DEXA. After ethanol fixation, cells were stained with crystal violet. (**A**) is a representative photograph of colonies of treated cells. (**B**) is a bar graph that depicts the means (±SD) of the colony counts. Wound healing assay (**C**–**D**) was performed on HepG2 treated similarly to the Clonogenic assay. Scratched areas were photographed after 24 h and compared to the corresponding zero time. “E” represents the area changes between untreated and treated cells

As treatment of MSCs with DEXA is known to promote their osteogenic differentiation, the activity of ALP enzyme (as a stemness marker), the expression of osteogenic genes (ALP, OPN, and RUNX2), and the deposition of intracellular calcium were assessed 24 h post-DEXA treatment (Fig. 4). Although lower concentrations of DEXA maintained cell stemness as indicated by the relative increase in the activity of ALP (Fig. 4A), a higher dose (1000 nM) led to a dramatic rise in ALP activity as an indication of the osteogenesis. In parallel, higher concentration (1000 nM) modulated the osteogenic-specific genes (Fig. 4A-4B). These changes were associated with the accumulation of intracellular calcium assessed by Alizarin Red S staining (Fig. 4C-5 F).

### Antimitotic effect of DEXA-MSC-S against hepatoma cells

Next, we aimed to investigate the healing potential of the DEXA-treated MSC-S, where two approaches were adopted. The first was to assess the antiproliferative effect on hepatoma cells *in vitro*. The second was to treat the ALF mice model with DEXA-MSCs-CM. The survivability of HepG2 cells and their ability to undergo unlimited division and colony formation were assessed by clonogenic assay, which demonstrated that HepG2 cells, grown in MSC/DEXA-CM for 48 h, significantly reduced the colony count, compared to the control well (Fig. 5A and 5B). A similar but to a lesser extent, changing pattern was observed in HepG2 cell migration, where similar treatments restricted cells’ *in vitro* migration (Fig. 5C-5E).

### Assessment of overproduction of ROS and glucose consumption

**Fig. 6:**
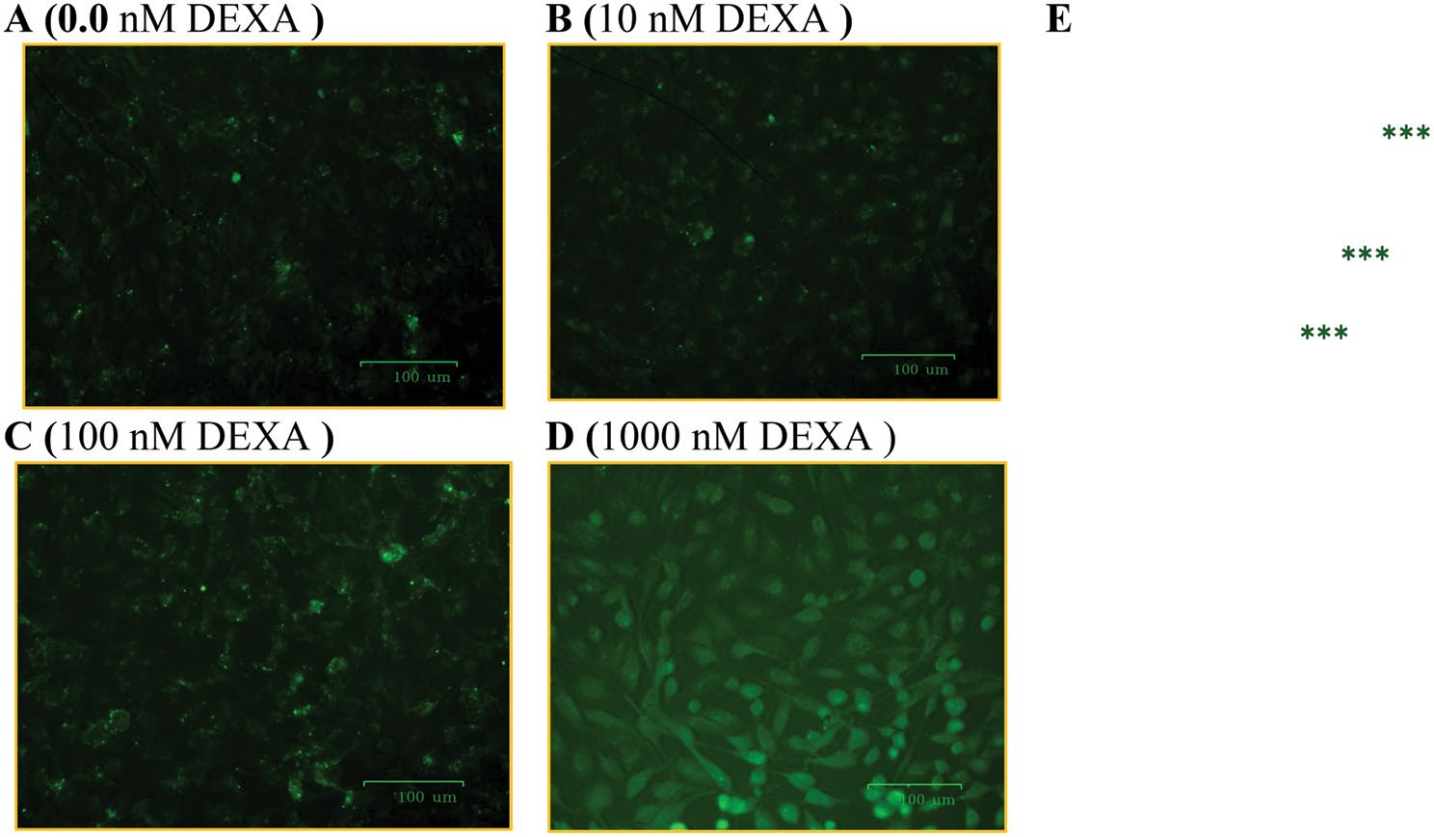
Fluorescent inverted micrographs demonstrate the assessment of cellular ROS by DCFH-DA. HepG2 cells were left untreated (**A**), treated with conditioned media derived from MSCs pretreated with 10, 100, or 1000 nM DEXA (**B**, **C**, and **D**, respectively). Bar graph (**E**) shows the integrated density of each treatment. (***): refer to significant (P < 0.001) difference between the indicated treatment versus control

**Fig. 7:**
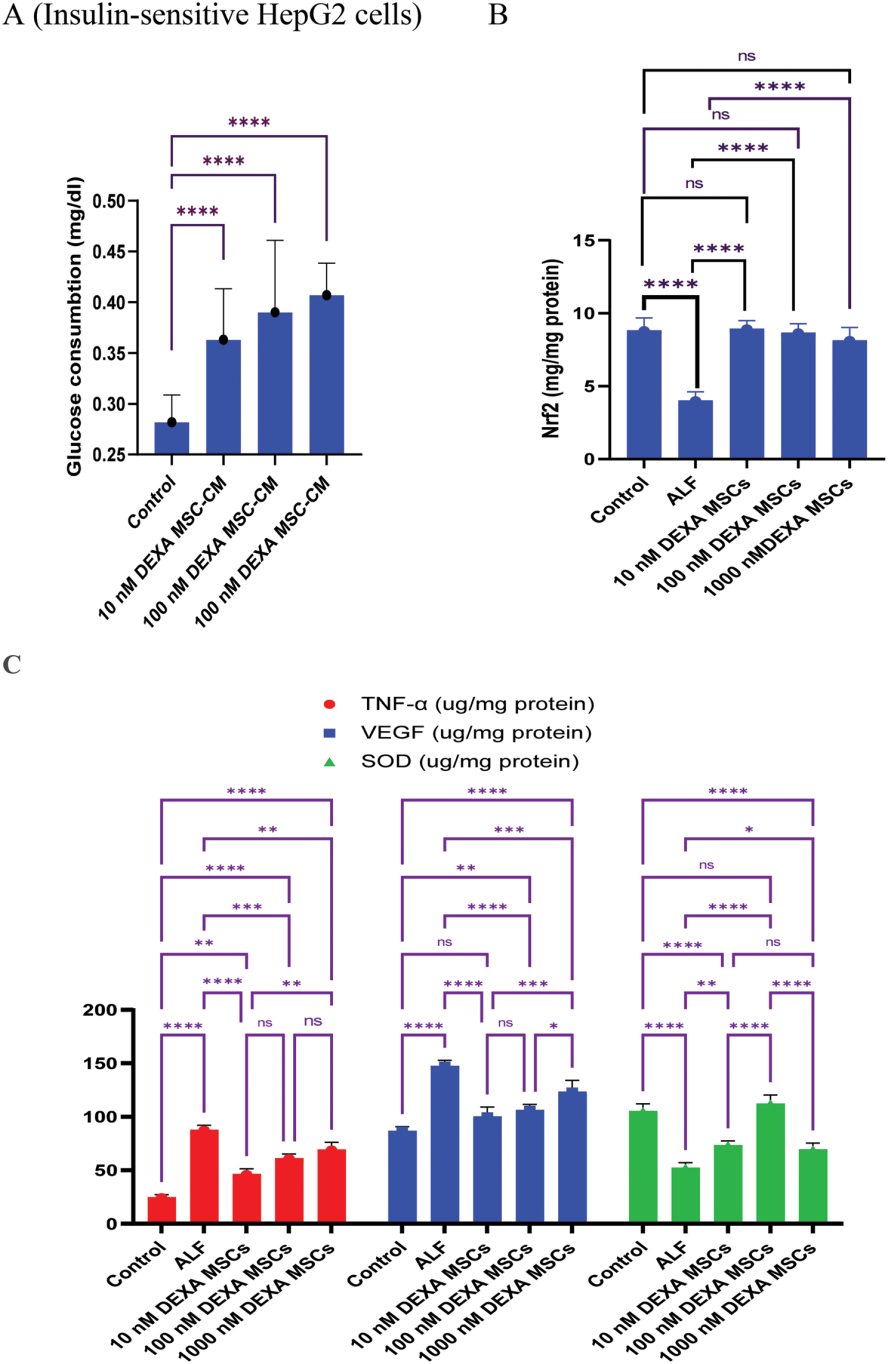
DEXA-treated BM-MSCs conditioned media decreased glucose uptake in hepatoma cells and reduced levels of hepatic inflammatory, angiogenic, and oxidative stress markers in mice. HepG2 cells treated with BM-MSC/DEXA-S significantly consumed less glucose compared with untreated control cells (P < 0.001) (**A**). Transfusion of BM-MSC-S in ALF mice variably reduced the inflammatory (TNF-α), and the angiogenic (VEGF) markers, and ameliorated the oxidative stress markers (Nrf2 and SOD) (**C**&**D**, respectively). Data are presented as mean (± SD)

The above findings were associated with the overproduction of cellular ROS (Fig. 6A-6E) in cells treated with the conditioned media derived from 1000 nM DEXA-modified MSCs (Fig. 6D-6E). Additionally, the glucose consumption by HepG2 cells reduced as the concentration of DEXA increased (Fig. 7A).

### Therapeutic effect of DEXA-MSCs-S on acute liver failure

**Fig. 8:**
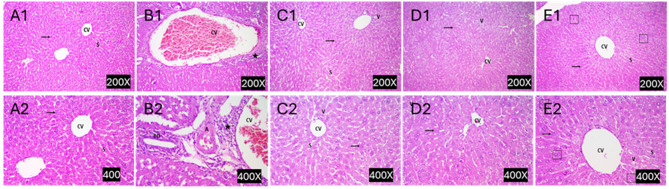
Histopathological assessment of DEXA-modified MSC conditioned media to treat APAP-induced acute liver failure in mice. Panels **A** and **B** are representative photomicrographs of H & E-stained sections of the liver, derived from healthy mice, APAP-induced ALF, and DEXA-treated MSC conditioned media. Pannels **C**, **D**, and **E** are stained sections derived from ALF mice which were treated with 10 nM, 100 nM, or 1000 nM DEXA-loaded MSC conditioned media, respectively (A1/2) show normal histological architecture, with radiating cords of hepatocytes surrounding the central vein (CV), normal hepatocytes with central rounded pale stained nucleus, and normal blood sinusoids (S). B1/B2 show disturbed histological architecture with dilated, congested central vein (CV), inflammatory infiltration (*). C1/C2 shows moderate improvement of histological architecture consisting of central vein (CV) with radiating cords of hepatocytes surrounding it, and normal hepatocytes with central, rounded, pale-stained nuclei. Also, a few hepatocytes contained cytoplasmic vacuoles (V) and dilated blood sinusoids (S). D1/D2 show mild improvement of histological architecture consisting of irregular central vein (CV) with radiating cords of hepatocytes, normal hepatocytes with central, rounded, pale-stained nucleus. E1/E2 show marked improvement of histological architecture, more or less similar to control group consisting of central vein (CV) with radiating cords of hepatocytes, normal hepatocytes with central rounded pale-stained nucleus (thin arrow), and normal blood sinusoids (S). Also, binucleated hepatocytes were seen (squares) with few cytoplasmic vacuoles

To investigate how far DEXA-MSC/-CM can restore liver integrity in the ALF mouse model, a panel of biomarkers was investigated in serum and liver homogenate derived from ALF mice. Initially, the liver function markers (ALT, ALP, γ-GT, and bilirubin) were increased dramatically due to APAP overdosing (group II) compared to healthy mice (Group I) (P < 0.001). Transfusion of DEXA-MSC-S significantly improved liver markers (Table 2). Furthermore, the hepatic levels of the antioxidant (SOD), the inflammatory (TNF-α) and the angiogenic (VEGF) makers were significantly improved in treated animals (Fig. 7C). These findings were associated with a notable improvement in the hepatic oxidative stress as indicated by the ameliorated levels of hepatic GSH, and MDA compared to the corresponding levels in ALF mice (Table 3). The improvements achieved via DEXA-MSC-S treatments were observed regardless of the initial concentrations of DEXA. Moreover, these alterations were associated with restoring the liver tissue integrity, marked by the clearance of the necrotic and inflammatory changes in the liver tissue (Fig. 8), one week after transfusion.

**Table 2:** Liver function serum biomarkers (ALT, ALP, gamma-GT, and total bilirubin) in acute liver failure mice transfused with DEXA-treated BM-MSC-conditioned media

	ALT (U/ml)	ALP (U/ml)	GGT (U/ml)	Bilirubin (mg/dl)
GP1 (Control)	23.30±5.10	65.66±5.03	0.48±0.03	1.19±0.09
GP2 (ALF)	1856.7±61.1 ***	623±37.58 ***	1.66±0.023 ***	1.99±0.09 ***
GP3 (10^−6^ DEXA MSCs-CM)	205.7±25.03 ***,♦♦♦	144.0±7.54 ***,♦♦♦	0.51±0.07 ns,♦♦♦	1.10±0.04 ns, ♦♦♦
GP4 (10^−7^DEXA MSCs-CM)	219.0±29.70 ***,♦♦♦	164.5±7.77 ***,♦♦♦	0.52±0.067 ns,♦♦♦	1.29±0.07 ns, ♦♦♦
GP5 (10^−8^ DEXA MSCs-CM)	206.0±56.10 ***,♦♦♦	208.7±18.71 ***,♦♦♦	0.64±0.06 ns,♦♦♦	1.28±0.03 ns,♦♦♦

*Abbreviations* ALF:  acute liver failure, ALT: alanine aminotransferase, ALP: Alkaline phosphatase, GGT: gamma glutamyl transferase and  Bili:  total bilirubin

**Table 3:** Transfusion of ALF mice with DEXA-treated BM-MSC-S ameliorated the levels of hepatic GSH and MDA

	Control	ALF	10^−6^ DEXA/ MSCs	10^−7^DEXA/ MSCs	10^−8^ DEXA/ MSCs
MDA (ng/mg protein)	1.633±0.15	4.26±0.55 ***	2.2±0.16 *** ♦♦♦	2.11±0.18 ** ♦♦♦	2.23±0.15 *** ♦♦♦
GSH (µM/gm tissue)	4.4±0.61	0.93±0.02 *** ♦♦♦	3.16±0.32 *** ♦♦♦	2.1±0.03 *** ♦♦♦	1.96±0.03 *** ♦♦♦

(*) comparison relative to the control

(♦ comparison relative to ALF group)

### Target fishing of DEXA and its metabolites

**Fig. 9:**
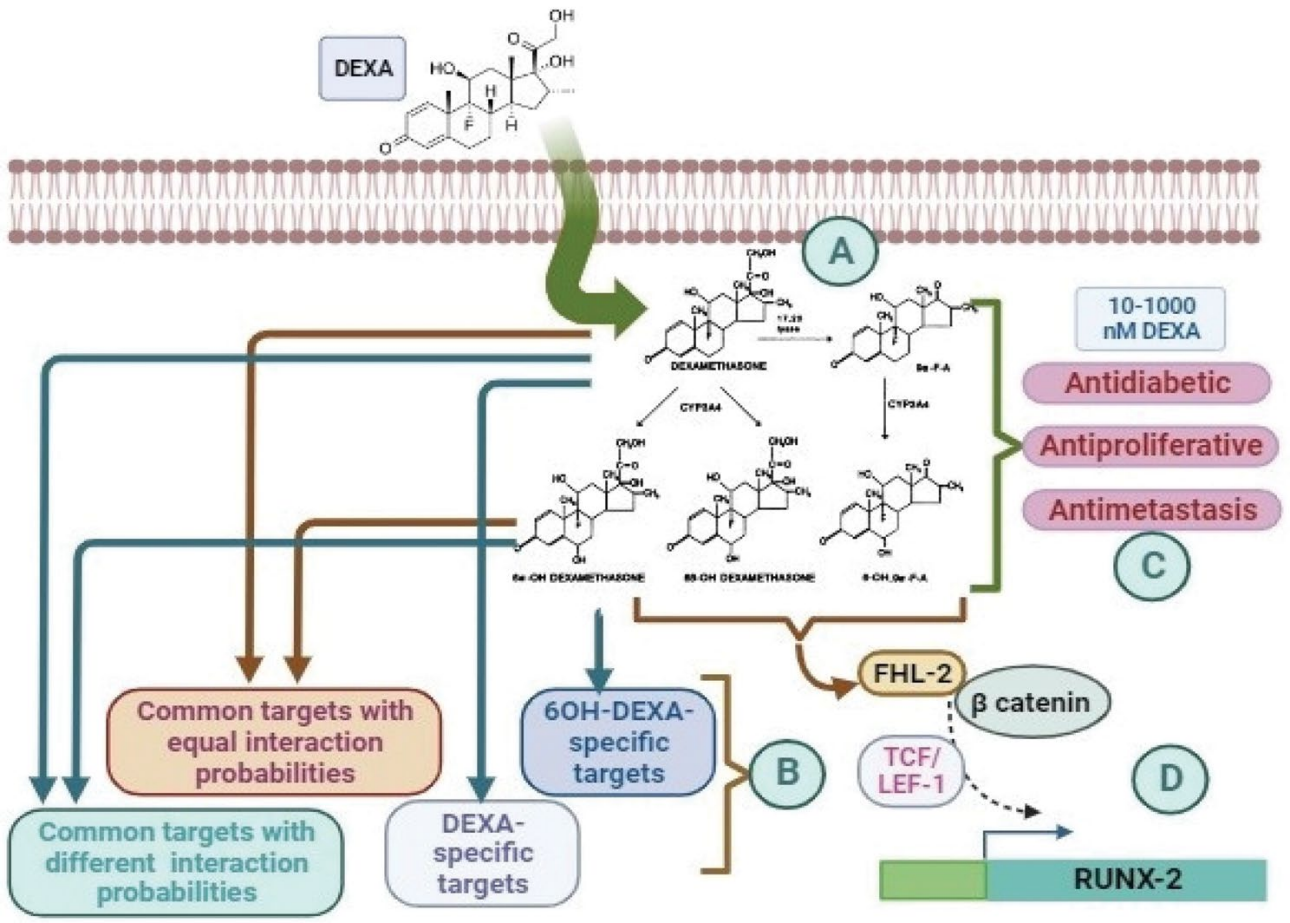
The Illustration depicts the metabolic conversion of DEXA in MSCs, its cellular effects, potential targeted proteins, and the upregulation of osteogenesis-related genes. After cell entry, DEXA is metabolized into 4 intermediates via CYP3A4 and lyase (**A**) [28]. Potential targets of DEXA and its major metabolic product (6βOH-DEXA) are predicted by NetTarget, where they are categorized into 4 sets of proteins (**B**). The cellular effects of submicromolar concentrations of DEXA (and/or its metabolites) are shown in (**C**). Higher concentrations of DEXA (1000 nM) led to the overexpression of the osteogenic-related gene (RUNX2) via the activation of β-catenin

As MSC mimics hepatic cells in the expression of some drug-metabolizing enzymes, prediction analysis was conducted to nominate the cellular targets of DEXA and its metabolites. This analysis identified three sets of proteins equivalently, differentially, or uniquely targeted by DEXA or its major metabolite (6β OH-DEXA) (Fig. 9, Tables 4&5).

**Table 4:** Baseline composition of MSC conditioned media and their regulatory functions

Regulatory components		Function
Proteins	*Growth factors (such as TGF-β, VEGF, HGF, FGF)*	Promote tissue repair and regeneration
	*Cytokines (such as L-10, TGF-β)*	Immunomodulatory effect
	*Enzymes (such as Metalloproteinases MMPs)*	Modulate the extracellular matrix
	*Various surface receptors*	Influence cell signaling and EV uptake
Lipids	*Phospholipids*	Form the lipid bilayer of the EV membrane.
	*Sphingolipids*	Involved in signaling pathways and membrane structure
	*Cholesterol*	Stability to the EV membrane
Nucleic Acids	*mRNA*	Encodes proteins that can be translated in recipient cells
	*microRNA (miRNA)*	Regulates gene expression post-transcriptionally
	*Long non-coding RNA*	Involved in regulating various cellular processes
	*DNA*	Occasionally present and may include mitochondrial DNA and genomic DNA fragments
Other Molecules	*Metabolites*	Influence recipient cell metabolism
	*Peptides*	Induce various biological activities
	*Exosomes-specific markers (CD9, CD63, CD81)*	Used to identify and isolate exosomes

## Discussion

In this study, we utilized nanomolar concentrations of dexamethasone in preconditioning BM-MSCs. Short-term treatment of BM-MSCs with 10, 100, or 1000 nM did not affect cell morphology and viability. Meanwhile, 1000 nM DEXA reduced the expression of MSC mesenchymal surface markers, increased the hematopoietic ones, and initiated osteogenic differentiation. Also, the conditioned media derived from cells pretreated with lower concentrations imposed antiproliferative and antimetastatic effects on hepatoma cells in vitro. More importantly, this formulated conditioned media effectively cured APAP-induced ALF in mice. The preconditioning drug we utilized (DEXA), is commonly used in clinical practice to inhibit the immune system and relieve the side effects of chemotherapy in cancer patients [28]. However, its pleiotropic effect was repeatedly reported due to its anti-inflammatory, immunosuppressive, and toxic effects [29, 30]. In another context, recent therapeutic regenerative trends nominated MSC-CM as a new generation of cell-free therapy. Previous studies suggested that conditioned media derived from normal MSCs demonstrate varying effects on the growth of HepG2. Some investigators revealed that MSC-S can enhance the growth and proliferation of HepG2 cells or inhibit their growth. This was attributed to the variability of biomolecules it contains. As Table 4 shows, MSCs-CM contains a mixture of growth factors, pro-survival signals, apoptotic proteins, and antiproliferative factors. In previous studies, DEXA has induced apoptosis in MSCs in a dose- and time-dependent manner [31], and its co-delivery with MSCs abolished their healing effect against liver fibrosis in mice [32]. These observations demonstrated the controversy about DEXA effect in MSCs that involve survival events, such as proliferation, differentiation, or apoptosis-mediated cell death [16]. Although the concentrations and the treatment time we utilized did not induce morphological or apoptotic changes, other studies found that treatment of MSCs with 1000 nM for longer periods (5 days) led to the overproduction of ROS, disrupted the mitochondrial dynamics, and developed apoptosis [17]. Also, short-term exposure maintained cell viability, even with higher concentrations (up to 15 µM) [33]. The obtained results revealed that 1000 nM DEXA led to the loss of the mesenchymal and triggered the osteogenic transition, on both the genomic and cellular aspects. These scenarios could be attributed to the direct effect of DEXA on the expression of FHL-2 that induces the expression of RUNX2 via the β-catenin pathway [34]. In addition to the time and concentration factors, the steroidal nature of DEXA facilitates its cellular uptake. Also, the expression of CYP3A4 in MSCs enhances the metabolic conversion of DEXA into 6β-hydroxy-dexamethasone (6βOH-DEXA) and 6α-hydroxydexamethaaone (6αOH-DEXA) [35] (Fig. 9A). Consequently, it is anticipated that there is a wide range of intracellular targets affected by DEXA and its metabolites. The similarity ensemble approach (SEA), we utilized, nominated many of these targets (Table 5). Moreover, target fishing identified three sets of intracellular proteins that equally, differentially, or uniquely interact with DEXA and/or 6βOH-DEXA (Table 6A, B, and C, respectively, (Fig. 9B). Furthermore, the downstream effect of DEXA and its metabolites may include signaling pathways like Wnt/β-catenin [36] and Notch signaling [37]. Thus, it is anticipated that treatment of MSCs with DEXA may modulate the basal content of their secretome. Also, it may enhance the anti-inflammatory role of cytokines, chemokines, and antiapoptotic factors secreted by MSCs [38, 39] to enhance the anti-inflammatory and antiproliferative effects. Furthermore, MSC-S usually contains soluble decoy receptors, such as TRAIL receptor CD264, which induce apoptosis or trap specific growth factors or cytokines required in cell survival [40]. These scenarios do not exclude the efflux of DEXA and its metabolites in the culture media. Previous studies demonstrated that MSCs loaded with paclitaxel can release the drug and inhibit the proliferation of mesothelioma cells [41]. Metabolically, DEXA reduced glucose consumption from the culture media, either due to the enhanced gluconeogenesis or the direct effect of DEXA on the expression of insulin receptors [42].

**Table 5:** Dexamethasone targets in human and mouse cells identified by the similarity ensemble approach (SEA) that relates proteins based upon the set-wise chemical similarity among their ligand. Maximum Tanimoto coefficients (maxTC) with smaller significance scores indicate a greater probability that the protein is a potential target of DEXA

	Target Name	Description	*P*-Value	MaxTC*
Human	NR3C1	Glucocorticoid receptor	1.918e-52	1.00
	NR3C2	Mineralocorticoid receptor	2.32e-06	1.00
	PGR	Progesterone receptor	9.879e-05	1.00
	AR	Androgen receptor	0.1817	1.00
	CYP2D6	Cytochrome P450 2D6	0.4004	1.00
	SERPINA6	Corticosteroid-binding globulin	1.118e-38	0.49
	SHBG	Sex hormone-binding globulin	1.79e-13	0.34
	GLUL	Glutamine synthetase	2.022e-13	0.43
	IL6	Interleukin-6	1.074e-10	0.43
	MMP1	Interstitial collagenase	1.564e-09	0.70
Rat	Nr3c1	Glucocorticoid receptor	2.827e-103	1.00
	Tat	Tyrosine aminotransferase	2.119e-177	0.68
	Fabp1	Fatty acid-binding protein, liver	2.022e-13	0.43

(*): MaxTC: Max Tanimoto coefficients

**Table 6:** Identified cellular targets of DEXA and 6βOH-DEXA demonstrate similar, close, or different interaction probabilities. Panel “A” includes common DEXA, and 6βOH-DEXA targets with similar interaction probabilities. Cellular proteins that are targeted differentially by 6β OH-DEXA, or DEXA are listed in panel B. Targets uniquely interacting with DEXA or its hydroxylated metabolite (6β OH-DEXA) are listed in panel C

	Uniprot _ID	Protein				Prob 6βOH-DEXA		Prob DEXA
**A**	P15207	Androgen receptor				1.0		1.0
	P04150	Glucocorticoid receptor				1.0		1.0
	P29477	Nitric oxide synthase, inducible				1.0		1.0
	P06401	Progesterone receptor				1.0		1.0
	P04058	Acetylcholinesterase				1.0		1.0
	P26358	DNA (cytosine-5)-methyltransferase 1				0.94		0.853
	P28566	5-hydroxytryptamine receptor 1E				0.934		0.994
	P23280	Carbonic anhydrase 6				0.588		0.314
**B**	P31213		3-oxo-5-alpha-steroid 4-dehydrogenase 2			0.757		1.0
	P08235		Mineralocorticoid receptor			0.646		1.0
	Q9QZN9		Cannabinoid receptor 2			0.537		0.999
	**6β OH-DEXA**				**DEXA**			
**C**	P24468		COUP transcription factor 2	0.881	P18405	3-oxo-5-alpha-steroid 4-dehydrogenase 1	1.0	
	O43353		Receptor-interacting serine/threonine-protein kinase 2	0.792	P05093	Steroid 17-alpha-hydroxylase/17,20 lyase	0.997	
	Q9NR96		Toll-like receptor 9	0.273	P22086	Alpha-2 C adrenergic receptor	0.851	
	Q00G26		Perilipin-5	0.269	O42713	Polyphenol oxidase 2	0.515	

DEXA: Dexamethasone; Prob: probability

**Fig. 10:**
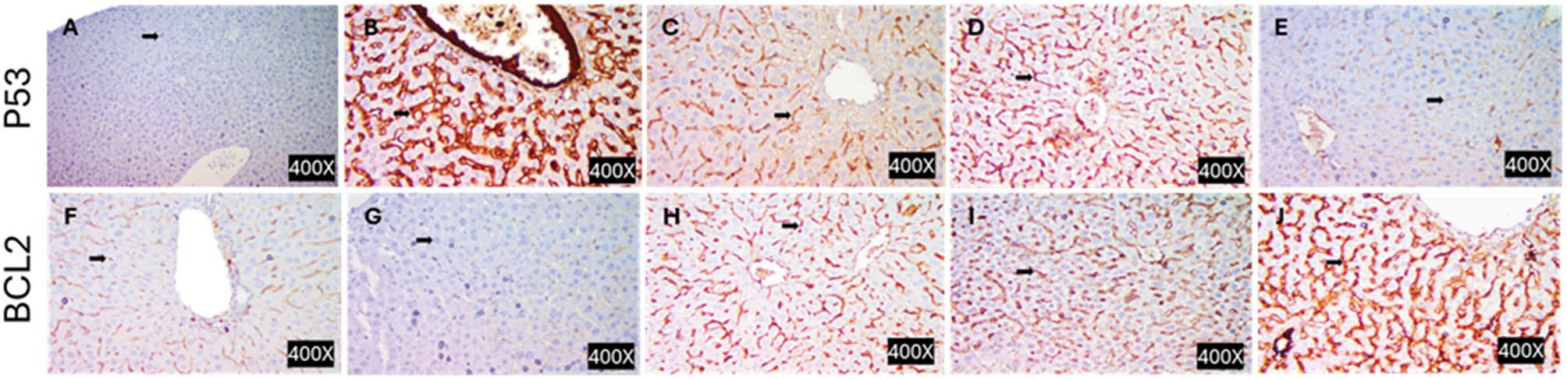
Representative photomicrographs of P53 immunohistochemical staining of liver sections from control mice showing negative reaction (**A**) and from ALF mice showing strong positive reaction (**B**). **C**, **D** and **E** represent sections from mice treated with 10, 100, 1000 nM DEXA-pretreated MSCs, showing mild, moderate and weak positive reactions, respectively. lower panels (**F**–**J**) are representative immunohistochemical staining photomicrographs of Bcl-2 antigen in liver sections counterstained with hematoxylin. (**F**) control showing mild positive reaction and (**G**) ALF mice, showing intense immunostaining of most of the hepatocytes. **H**, **I**, and **J** are sections from mice treated with 10, 100, and 1000 nM DEXA-modified MSCs, showing moderate, mild, and strong positive reactions, respectively. (magnification 400X)

To explore the *in vivo* effect of DEXA-MSC-CM, we established a mouse model of APAP-induced ALF. While limited studies have explored the therapeutic potential of MSCs [21, 43], we adopted a cell-free approach. This strategy provides more advantages over cell-based treatment, as it avoids the complications of MSC transfusion, including hypoxemia, misshoming, and host-related inflammatory reactions. It is anticipated that MSC-excreted cytokines, including IL-10, IL-4, hepatocyte growth factor (HGF), PGE2, tumor necrosis factor-inducible gene-6 (TSG-6), and heme oxygenase-1 (HO-1), stand behind the regression of APAP-induced apoptotic changes in the ALF model as indicated by the improvement of hepatic P53 and BCl-2 levels (Fig. 10). IL-10, for example, improved mitochondrial damage of hepatocytes, via the STAT3 signaling pathway [44]. Moreover, HGF attenuated hepatocyte necrosis, promoted proliferation [45], and reduced macrophage infiltration [46]. In a similar context, our previous work treatment of premature ovarian failure (POF) with the conditioned media of hypomethylated MSCs demonstrated more healing effects than MSC transplantation [47]

## Conclusion

This study presents a cell-free regenerative therapeutic approach and suggests that short-term exposure of BM-MSCs to nanomolar concentrations of dexamethasone maintains MSC viability and MSC markers. The data revealed that the DEXA-modulated conditioned media exerted antiproliferative effects in hepatoma cells. In vivo, DEXA-modulated conditioned media relieved the symptoms associated with acute liver failure and resolved hepatic necrosis. Further studies are suggested to explore the direct effect of DEXA, and its metabolites on MSC conditioned media components to explain the associated paracrine healing potential of MSC-S. Although ALF is a good hepatic inflammatory model, long-term *in vivo* assessments, such as liver fibrosis, cirrhosis, and HCC, are required to evaluate the efficacy of this approach.

## Data Availability

The data are available from the corresponding author upon reasonable request.
